# Creating three dimensional models of Alzheimer’s disease

**DOI:** 10.1186/s41205-017-0020-5

**Published:** 2017-11-21

**Authors:** Matthew Marks, Amy Alexander, Joseph Matsumoto, Jane Matsumoto, Jonathan Morris, Ronald Petersen, Clifford Jack, Tatsuya Oishi, David Jones

**Affiliations:** 1 Departments of Neurology, Mayo Clinic, Rochester, MN 55905 USA; 2 Department of Radiology, Mayo Clinic, Rochester, MN 55905 USA

**Keywords:** Hippocampus, 3D printing, Alzheimer’s disease, Dementia, Brain, Additive manufacturing

## Abstract

**Background:**

Alzheimer’s disease prevalence will reach epidemic proportions in coming decades. There is a need for impactful educational materials to help patients, families, medical practitioners, and policy makers understand the nature and impact of the disease. Defining an effective workflow to create such models from existing segmentation tools will be a valuable contribution in creating these patient-specific models.

**Results:**

A step-by-step workflow was developed and used to take patients’ Digital Imaging and Computing in Medicine magnetic resonance brain images through a process resulting in illustrative 3D–printed brain and hippocampus models that clearly demonstrate the progressive degenerative changes caused by Alzheimer’s disease. We outline the specific technical steps of auto-segmentation, manual smoothing, Standard Triangle Language file customization, and 3D printing used to create these models.

**Conclusions:**

Our explicated workflow can create effective models of Alzheimer’s brains that can be used in patient education, medical education, and policy forums.

## Background

Alzheimer’s disease is a neurodegenerative disease causing progressive and disabling cognitive decline. As populations age, the prevalence of Alzheimer’s disease is increasing dramatically. Currently, in the United States, 5.5 million people are afflicted with Alzheimer’s disease, with this number estimated to rise to 15 million by 2050 [1].

Given the rising magnitude of this public health problem, educational materials that visualize the progressive impact of Alzheimer’s disease would help patients, families, and medical practitioners understand the diagnosis and disease process. Computerized tomographic (CT), magnetic resonance imaging (MRI) and positron emission tomographic (PET) scans are often used for such purpose but may be difficult for the non-radiologist to interpret. We believe three-dimensional anatomic models are ideal for education and counseling, enabling individuals to hold the Alzheimer’s process in their hands, touching and visualizing its impact.

**Fig. 1:**
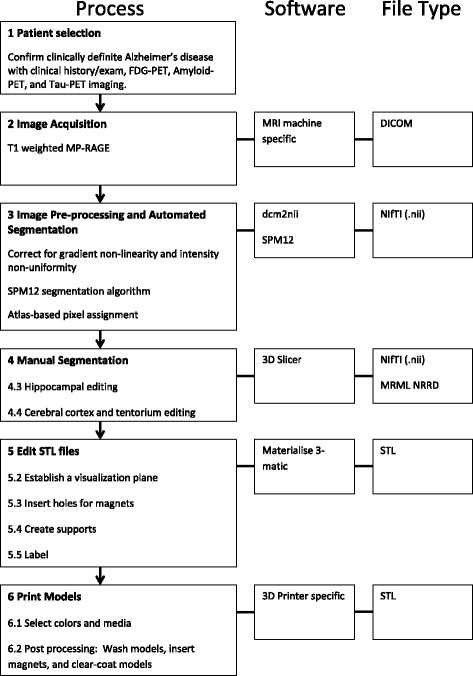
Flowchart of Workflow for 3D models of Alzheimer’s disease. Abbreviations listed in text

Anatomically, Alzheimer’s disease affects the cerebral cortex and mesial temporal lobe structures, most prominently the hippocampus. Reduced hippocampal volume as measured by MRI has been long recognized as a biomarker for Alzheimer’s disease [2]. Other radiologic biomarkers include amyloid-specific imaging (Pittsburgh compound B (PiB)-PET imaging), tau-PET imaging, [(18)F]-fluorodeoxyglucose (FDG)-PET and measures of cortical thickness or brain volume [3]. We chose to build models that emphasized progressive loss of cortical and hippocampal volume. Modeling the hippocampus posed challenges because segmentation of this structure is difficult, and displaying its anatomy requires exposure of the deep mesial brain. In this article we outline a detailed workflow that allows construction of individualized, three-dimensional models of a normal brain and those afflicted with Alzheimer’s disease (Fig. 1).

## Methods

### Selection of subjects

#### Database

Subjects were drawn from the database of the Alzheimer’s disease Research Center and the Mayo Clinic Study of Aging at the Mayo Clinic, Rochester, Minnesota [4]. The Mayo Clinic and Olmsted Medical Center Institutional Review Boards approved these studies and written informed consent was obtained from all participants and/or their qualified representative.

#### Subject characteristics

We chose one subject in each of the following clinical categories: normal control, clinically-mild Alzheimer’s dementia, clinically-moderate Alzheimer’s dementia, clinically severe Alzheimer’s dementia (Severe-1), and Alzheimer’s dementia with radiologically severe hippocampal atrophy (Severe-2). Detailed clinical characterization of the subjects is contained in Table 1.

**Table 1:** Clinical characteristics of subjects

Classification	Age/Sex	Symptoms	Living arrangement	DRS	FDG-PET	PiB-PET	Tau-PET
Normal	54/F	None	Independent	143 (94%)	(−)	(−)	(−)
Clinically-Mild AD	78/M	Mild memory dysfunction. Rarely gets lost driving	Independent	120 (1%)	Precuneus hypometabolism	(+)	(+)
Clinically-Moderate AD	74/F	Memory poor. Gets lost. Reduced daily activity.	Assisted Living	96 (<1%)	Bilateral parietotemporal hypometabolism	(+)	(+)
Clinically-Severe AD (Severe-1)	84/M	Doesn’t recognize family. Needs help dressing and bathing. Incontinent.	Nursing home	83 (<1%)	Bilateral frontal, parietal, temporal hypometabolism	(+)	(+)
AD with radiologically severe hippocampal atrophy (Severe-2)	81/M	Severe memory loss. Gets lost driving and in home.	Independent	112 (<1%)	Bilateral parietotemporal, lateral occipital, precuneus, and posterior cingulate hypometabolism	(+)	(+)

*Abbreviations*: *AD* alzheimer’s disease, *F* female, *M* male, *DRS* dementia rating scale (144 point scale, lower scores indicate poorer cognitive functioning, age-related normal percentile indicated in parentheses [16]), *FDG-PET* [(18) F]-fluorodeoxyglucose positron emission tomography, *PiB-PET* Pittsburgh compound B positon emission tomography, *Tau-PET* tau positon emission tomography, (−) = negative scan results, (+) = positive scan results

### Image acquisition

T1-weighted structural MRI images were acquired on a General Electric (GE) 3 T scanner with a sagittal 3D magnetization-prepared rapid acquisition gradient-recalled echo (MP-RAGE) sequence. Repetition time was ≈ 2300 ms, echo time ≈ 3 ms, inversion time ≈900 ms, and voxel dimensions were ≈ 1.20 × 1.015 × 1.015 mm.

### Image pre-processing and automated segmentation

Digital Imaging and Computing in Medicine **(**DICOM) files from the GE scanner were converted to Neuroimaging Informatics Technology Initiative (NIfTI, file extension .nii) files through the freeware utility dcm2nii (Neuroimaging Informatics Tools and Resources Clearinghouse, www.nitrc.org). All T1-weighted MRI scans underwent correction for gradient non-linearity and intensity non-uniformity prior to being processed using the SPM12 (http://www.fil.ion.ucl.ac.uk/spm/software/spm12/) unified segmentation and normalization algorithm [5, 6]. A custom template with six tissue classes was used for unified segmentation and normalization. The inverse warps created during normalization were used to back propagate regions of interest (ROIs) from a customized version of the automated anatomical labeling atlas into the native space of the original structural MRI [7]. The native space white matter and gray matter segmentations were combined and used as an inclusive brain tissue mask to extract the brain anatomy of interest from the preprocessed T1-weighted MRI in native space. The back propagated hippocampal ROI was then masked by the gray matter tissue segmentation to exclude voxels in the ROI that did not fall within segmented gray matter.

### Manual segmentation

*4.1 File Transfer:* The auto-segmentations of the hippocampus, gray matter, and white matter were loaded as NIfTI files into 3D Slicer (https://www.slicer.org) [8]. The software created Medical Reality Markup Language (MRML) and Nearly Raw Raster Data (NRRD) files as part of the segmentation process, which was completed by creating and exporting Standard Triangle Language (STL) files.

#### Hippocampal segmentation assessment

**Fig. 2:**
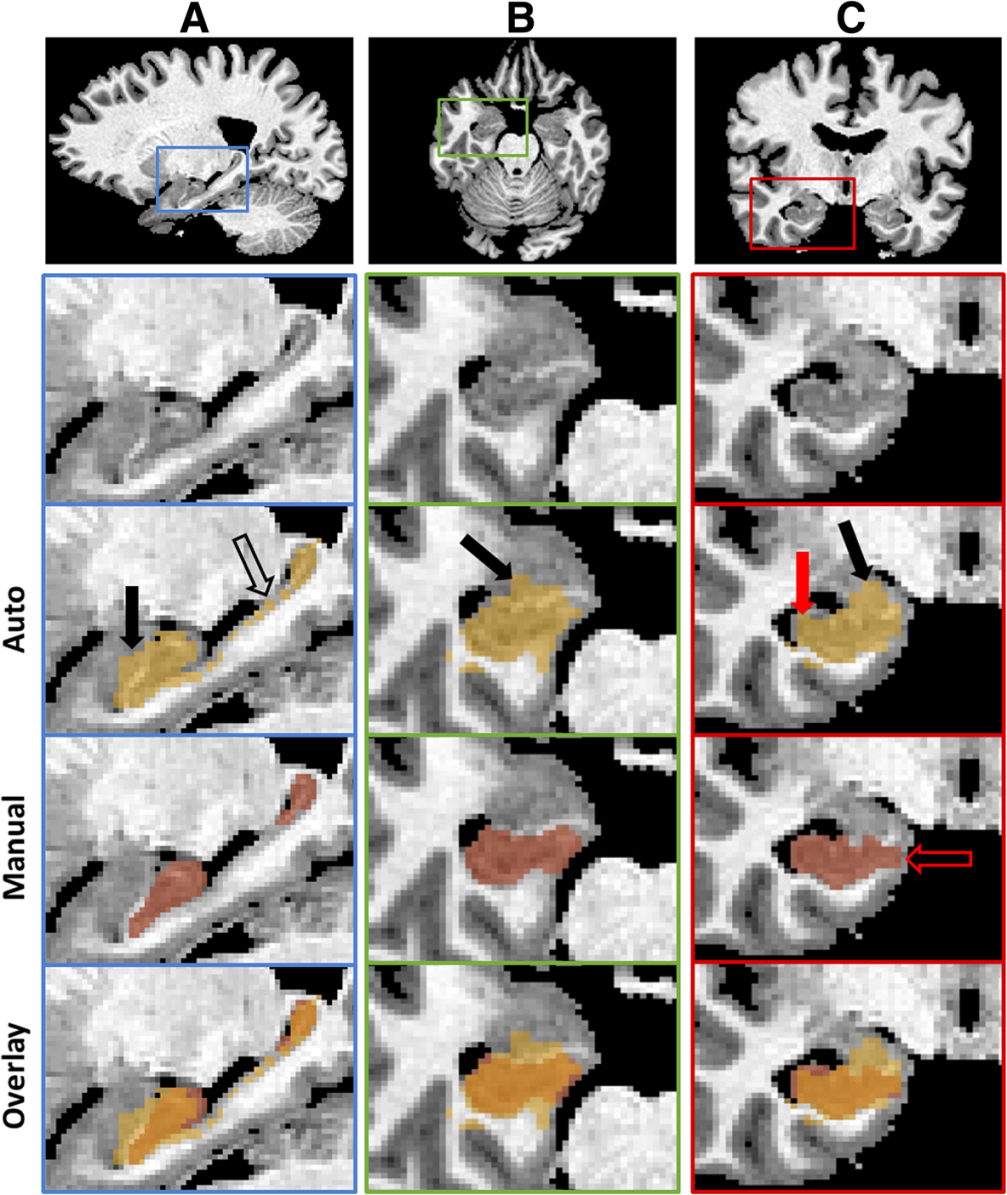
Manual segmentation and smoothing of the hippocampus. Example of manual editing of the atlas-based auto-segmentation at the head of the hippocampus. Manual segmentation is performed with images in the sagittal (**a**), axial (**b**), and coronal (**c**) planes. Top row shows raw images with colored squares magnified in second row. Third row shows pixels assigned as hippocampus in gold. Fourth row shows final manual segmentation in red. Fifth row shows both auto-segmentation and manual segmentation with overlap in orange. Solid black arrows show areas where amygdala is auto-segmented as hippocampus. Solid red arrow shows area where the lateral border of the hippocampus is not smoothly assigned in auto-segmentation. Open black arrow shows extra pixels assigned to the dorsal hippocampus border in the auto-segmentation that are outside of hippocampus. Open red arrow shows the manual editing of the medial hippocampus border that included the subiculum

Preliminary STL files of the auto-segmented hippocampus and combined gray and white matter were created and used for an initial test print. Surfaces of the hippocampal models revealed ridges and irregular spikes or nodules that were not seen in the reference anatomic atlas [9] (Fig. 2). Analysis of the digital 3D renderings and the segmented MRI images indicated that nodules were often due to the printing of “stray” aggregations of misassigned pixels in nearby grey matter structures, and ridges were due to incomplete or “stray” assignment along hippocampal borders (Fig. 2). Likewise, the combined gray and white matter model included some surface irregularities, suggesting the need for further editing of the auto-segmentation.

#### Manual hippocampal segmentation editing

To correct for this loss of fidelity, a manual correction protocol was developed. Within 3D Slicer an individual hippocampus was displayed in three orthogonal planes with a sagittal plane along the anterior commissure-posterior commissure line, a coronal plane and an axial plane. The goal was to preserve the broad outlines of the auto-segmentation, but to improve the surface smoothness. The method used conformed to the guidelines outlined in the EADC-ADNI Harmonized Protocol for Manual Hippocampal Segmentation User Manual (HarP); however, in a deviation from the protocol, segmentation was initially performed in the sagittal plane (rather than the coronal plane) before further editing in the coronal and axial planes to check and refine the segmentation [10, 11]. Editing was done as follows:

#### Separating the amygdala from the hippocampal head

A small band of white matter often demarcates the amygdala from the hippocampal head. Errors arose when auto-segmentation included pixels on the amygdala side of this band (Fig. 2). This border was traced smoothly using the border of hippocampal grey matter with the white matter band. In doing this, portions of the alveus were likely eliminated; however, this technique resulted in satisfactory smoothing of hippocampal grey matter at the head of the hippocampus.

#### Smoothing the dorsal border of the hippocampal body

**Fig. 3:**
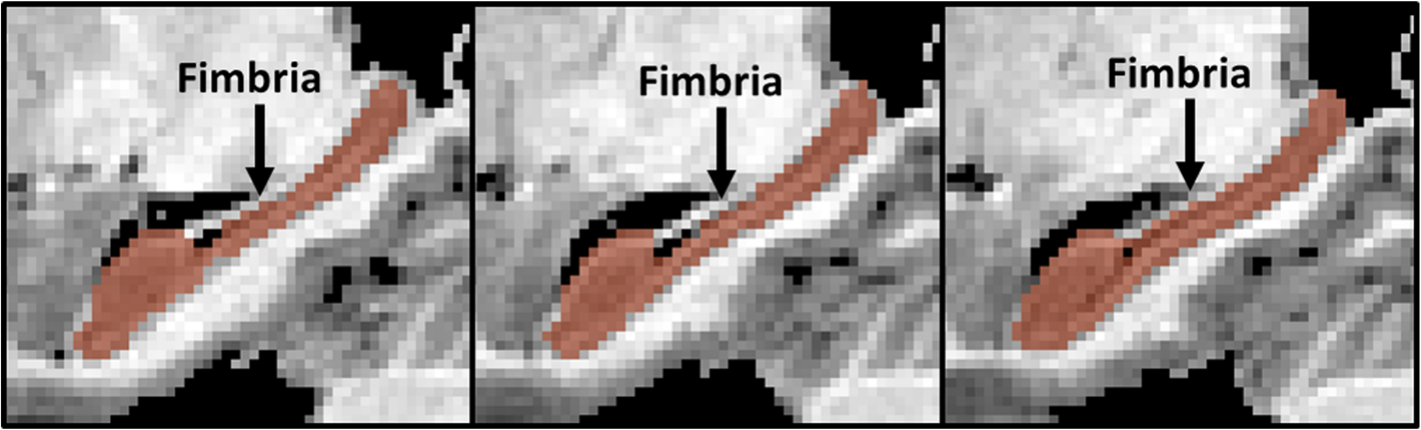
Exclusion of the fimbria. Sagittal sections: each image from left to right moves one slice from medial to lateral (1 mm slice thickness). The fimbria is seen as a dorsal structure of mixed intensity. At the resolution of 1 cubic millimeter its margins could not be smoothly segmented, and it was excluded from the manual segmentation

Frequent discontinuities arose along the dorsal margin of the auto-segmented hippocampal body (Fig. 2). These were often due to gaps along this border or to misassignment of areas suspected to be choroid plexus. Editing was performed initially in the sagittal plane with frequent re-evaluation in the coronal and axial planes. The fimbria was seen as an often ill-defined mixed intensity structure projecting dorsally above the medial body of the hippocampus (Fig. 3). At one millimeter pixel resolution this structure could not be smoothly segmented and was eliminated. In this process, editing decisions were made pixel-by-pixel at one millimeter pixel resolution. Smoothness and sharp changes in intensity values were employed as criteria in decision-making using subjective optimization (Fig. 2).

#### Editing of the medial border

The edge of the subiculum and the lateral ventricle offers a smooth, high contrast border for segmenting. This was outlined as the most medial aspect of the hippocampus, thus including the subiculum in concert with HarP instructions [10, 11]. An arbitrary horizontal line divided the subiculum from the parahippocampal gyrus (Fig. 2).

#### Smoothing the ventral border of the hippocampus

In most cases, the ventral boundary needed little editing. Grey matter of the hippocampus was separated from white matter of the parahippocampal gyrus, creating a smooth margin (Fig. 2).

#### Editing the lateral border of the hippocampus

A clear contrast between lateral ventricle and hippocampus offers a smooth segmentation border. At this segmentation border the overlying white matter of the alveus was included (Fig. 2).

#### Defining the rostral border of the hippocampus

Manual editing was continued to the trigone of the lateral ventricle where the hippocampus is abutted by and distinguishable from the indusium griseum.

**Fig. 4:**
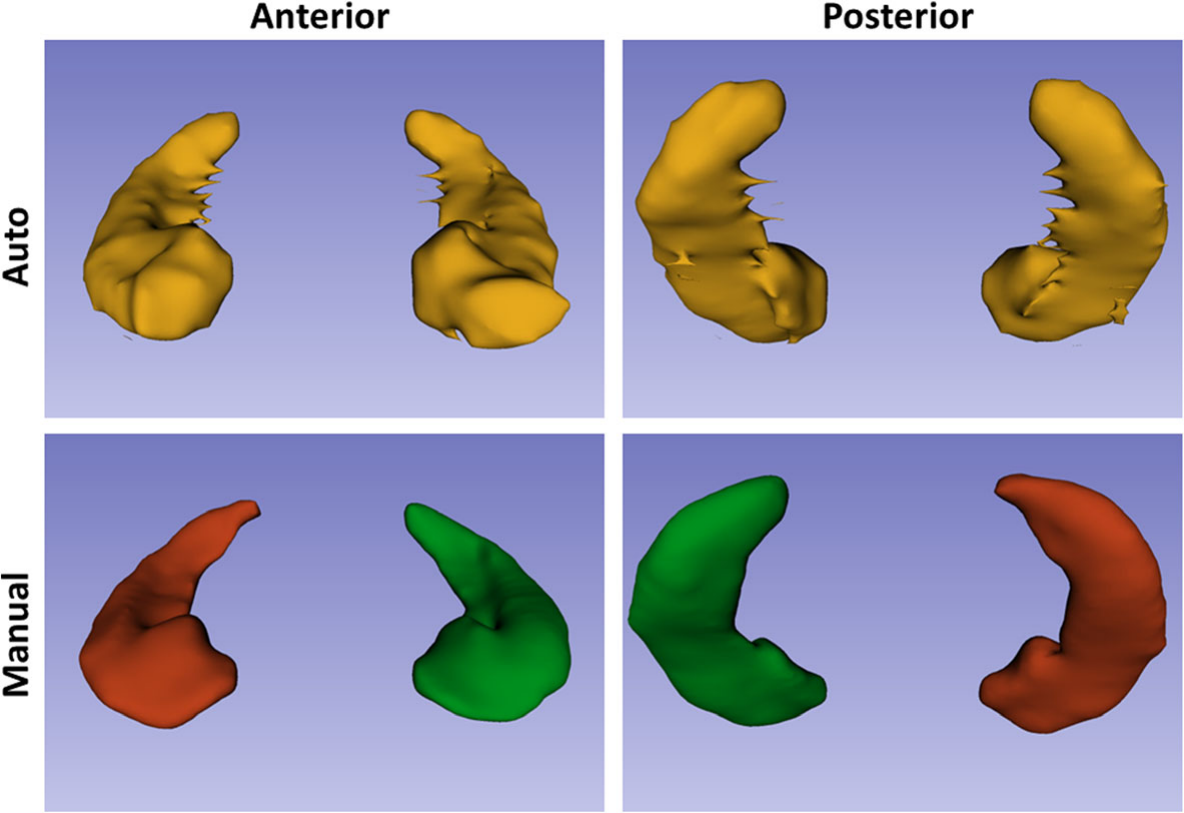
Comparison of auto- and manual segmentation. STL files produced by auto-segmentation (top row) and manual segmentation (bottom row). Left column views the hippocampi from anterior and dorsal. Right column shows view from posterior and ventral

3D renderings of the hippocampus were examined and compared to the initial auto-segmentation. Manual editing resulted in smoother renderings, predominately along the dorsal margins of the hippocampus, and more accurate division of the hippocampus from the amygdala (Fig. 4).

### Manual editing of the cerebral cortex

#### Editing the cortical surface

**Fig. 5:**
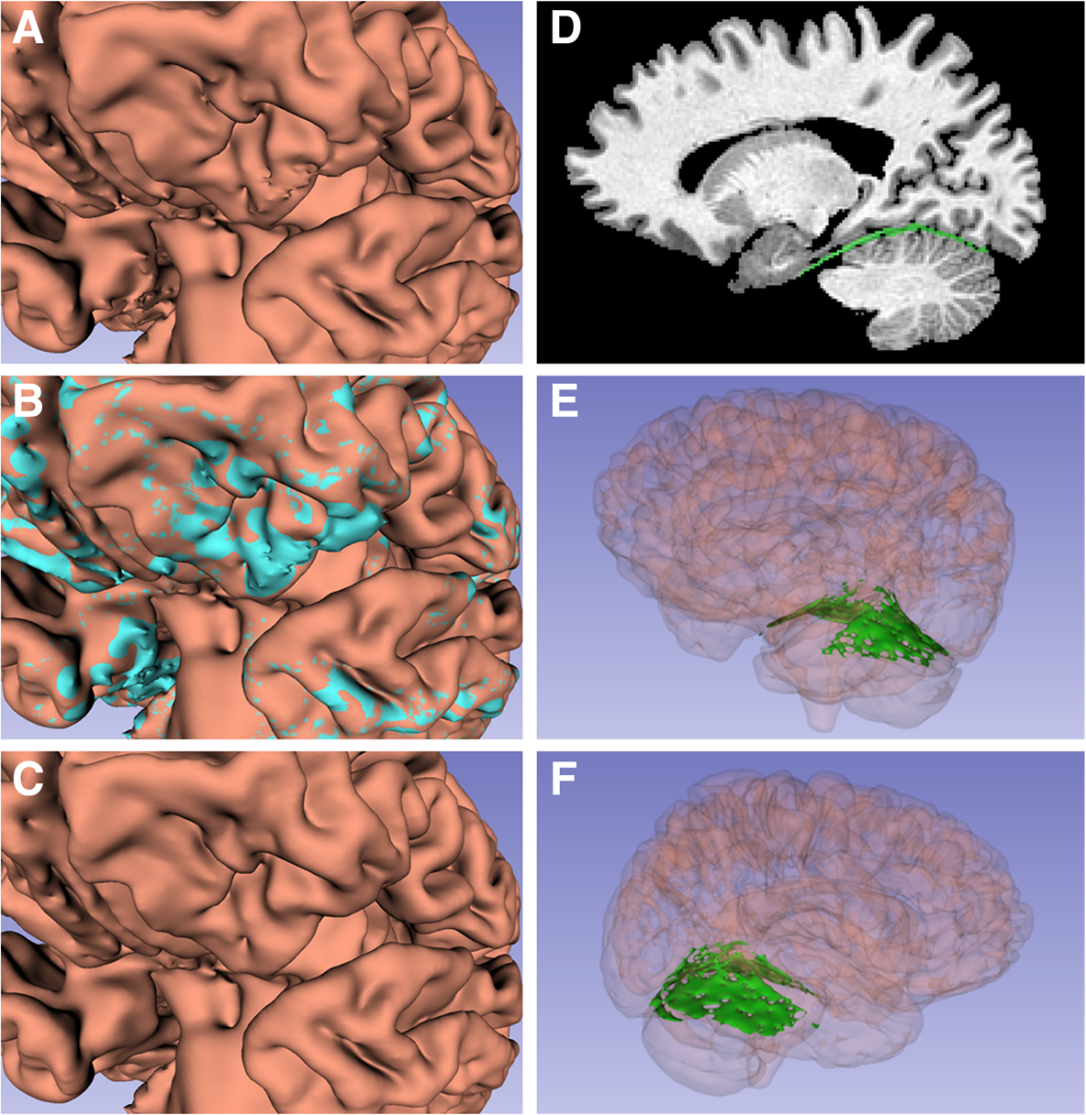
Manual smoothing of cortical surface and exclusion of the tentorium Panel **a**: White and gray matter imported from SPM included protrusions and nodules that required editing. These bumps likely represent dura, cortical veins and arteries, and sinuses. Panel **b**: Protrusions, manually highlighted in turquoise, are removed to produce a smooth cortical surface shown in Panel **c**. Panel **d**: Representative sagittal slice showing the manual segmentation of the tentorium, which is subtracted from the final brain segmentation. 3D rending of the tentorium from the left (Panel **e**) and right (Panel **f**), with the rest of the brain transparent

Spikes, protrusions, and various structures inconsistent with a smooth cortical surface were frequently included in the gray matter auto-segmentation. These structures likely represented dura and cortical arteries, veins, and sinuses and were thus manually eliminated (Fig. 5).

#### Removing the tentorium cerebri

The tentorium cerebri was included in the auto-segmentation, obscuring the separation of the cerebral cortex from the cerebellum. This was manually edited by creating a separate segmentation of the tentorium and subtracting it from the overall brain segmentation (Fig. 5).

### Creating and editing the STL file

#### File management

Once the edited segmentations produced satisfactory preliminary 3D renderings, they were converted into STL files and exported to computer-aided design (CAD) software (Materialise 3-Matic, Plymouth, MI). Separate STL files were created for the right and left hippocampus and the rest of the combined brain matter.

#### Establishing a visualization plane

**Fig. 6:**
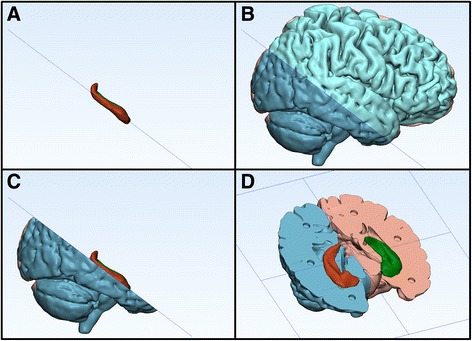
Cutting plane used to achieve better visibility of the hippocampus. Panel **a**: Plane created parallel to longest axis of hippocampus. Panel **b**: Both brain hemispheres split by plane, producing quadrants. Panels **c** and **d**: Removing ventral/anterior quadrants gives a clear view of the hippocampi, which are permanently affixed to the ventral/posterior quadrants

The hippocampus is deep within the brain, distant from the surface. The cloudiness of the semi-transparent material available for printing and the distortion of light caused by the curvature of the brain surface prevent meaningful visualization of the hippocampus when printed fully embedded within the rest of the gray and white matter. To address this, a cutting plane was defined parallel to the long axis of the hippocampi and used to divide the brain into dorsal and ventral sections that could be separated to reveal the hippocampus (Fig. 6). Another plane defined by the midline of the brain was used to split the model into left and right hemispheres, resulting in a total of four brain quadrants, with left and right hippocampus permanently affixed to the corresponding ventral quadrant.

#### Inserting holes for magnets

Holes were created for magnets that would hold the printed brain quadrants together but allow easy disassembly for viewing the hippocampus (Fig. 6).

#### Creating supports

Small supports were added as needed to reinforce the tip of the hippocampus, the cerebellum, and fragile areas of the cortex.

#### Labeling

An inconspicuous label was placed on the posterior side of the brain stem of each model.

### Printing the STL files and post-processing

#### Printer and media

Models were printed on a Stratasys Objet500 Connex3 (Stratasys, Eden Prairie, MN). The hippocampi were printed in shades ranging from green to blue using a mixture of VeroCyan and VeroYellow, which are opaque. To allow better visualization of the hippocampus within the whole brain, VeroClear, which is semi-transparent, was used for the surrounding brain tissue. The print time for a model was 15–20 h depending on brain volume.

#### Post-processing

The models were separated from the support material using small picks, a power washer, and a lye bath. Neodymium magnets (K&J Magnetics, Pipersville, PA) were attached to the models using cyanoacrylate glue. The transparency of the VeroClear was improved by applying a coating of Omni MC260 Quick Clear (PPG Industries, Pittsburgh, PA) to the surface.

#### Data analysis

Volumes of the auto-segmented hippocampi and manually edited hippocampi were generated and normalized to total intracranial volume. Hippocampal volumes from the two methods were compared by a single paired Student’s t-test and Pearson’s linear correlation. A *p*-value of <.05 was designated as significant.

## Results

**Fig. 7:**
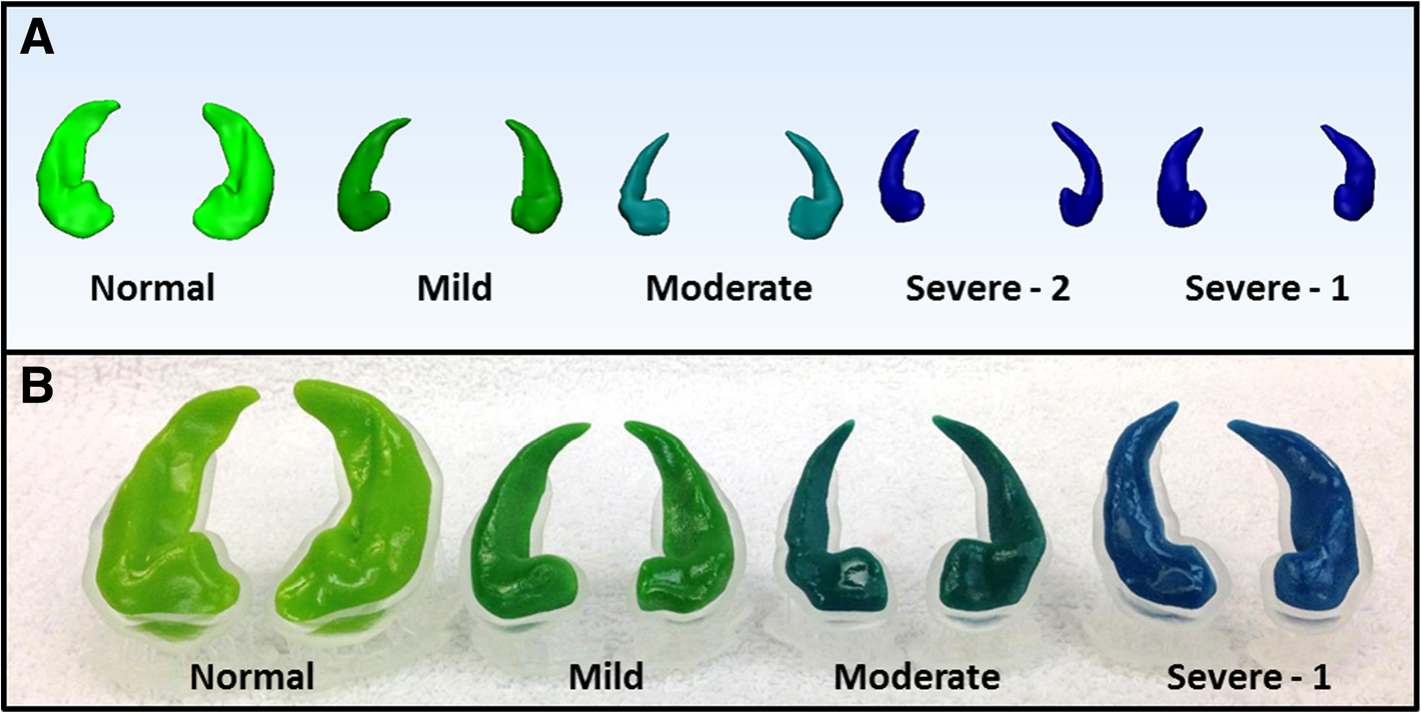
Hippocampus STL files and 3D–printed models. Row **a**: Hippocampal STL files are displayed with increasing disease severity from normal to severe Alzheimer’s disease moving from left to right. Row **b**: Final printed models corresponding to the normal hippocampus and three stages of Alzheimer’s disease severity and displayed on custom printed stands. Hippocampal size declines with both age and Alzheimer’s disease severity, and the models clearly demonstrate these effects. Radiologically severe model not available for photo

**Fig. 8:**
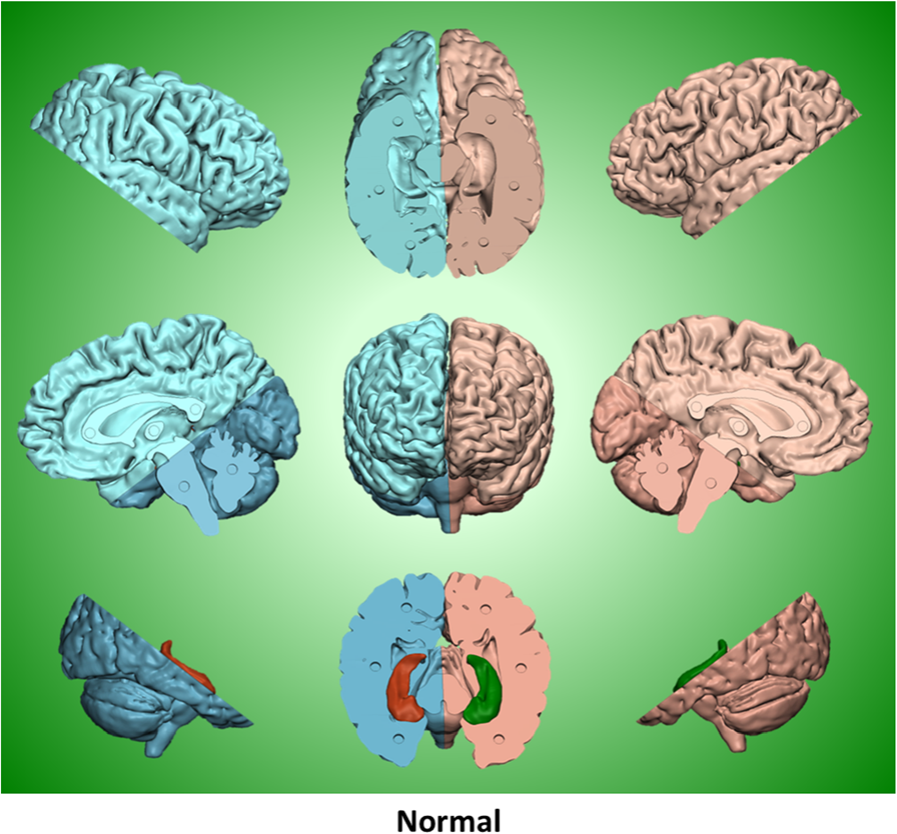
Normal brain STL files. The final STL files of the normal brain are displayed showing the cutting planes, sectional breakdown and magnet holes. The following STL images in Figs. 7 and 8 and the photographs in Figs. 9 and 10 are all displayed at the same scale, so the contrast in size between the normal and severe Alzheimer’s brains pictured here is representative of the disparity displayed by the physical models themselves

**Fig. 9:**
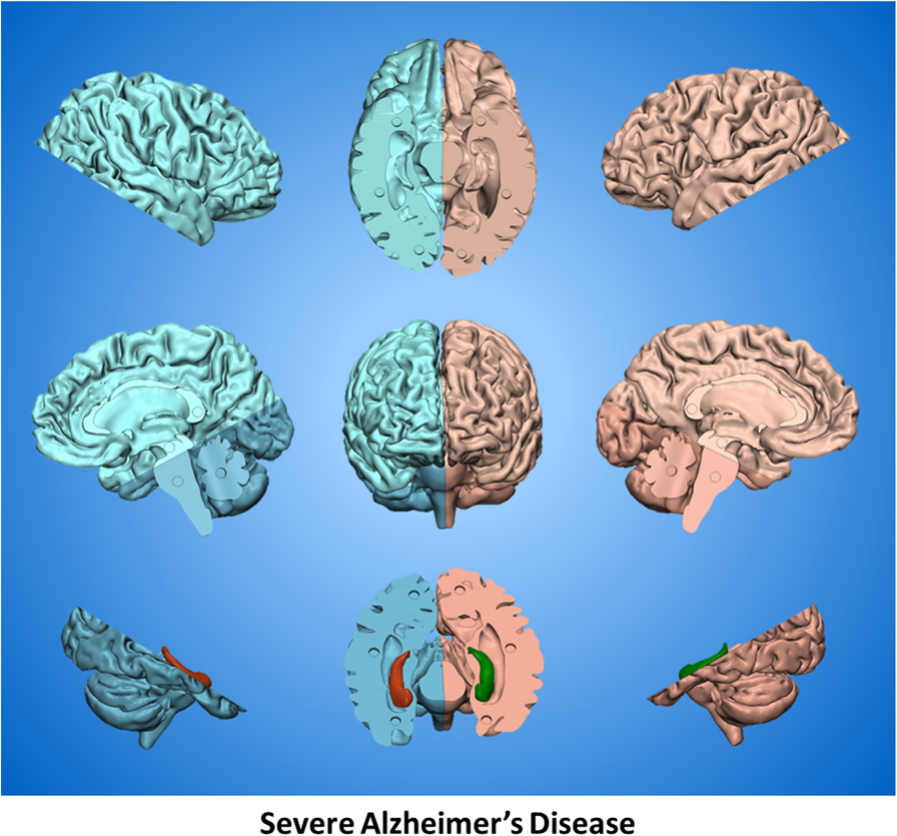
Alzheimer’s brain STL files. The final STL files for the clinically severe (Severe-1) Alzheimer’s brain

**Fig. 10:**
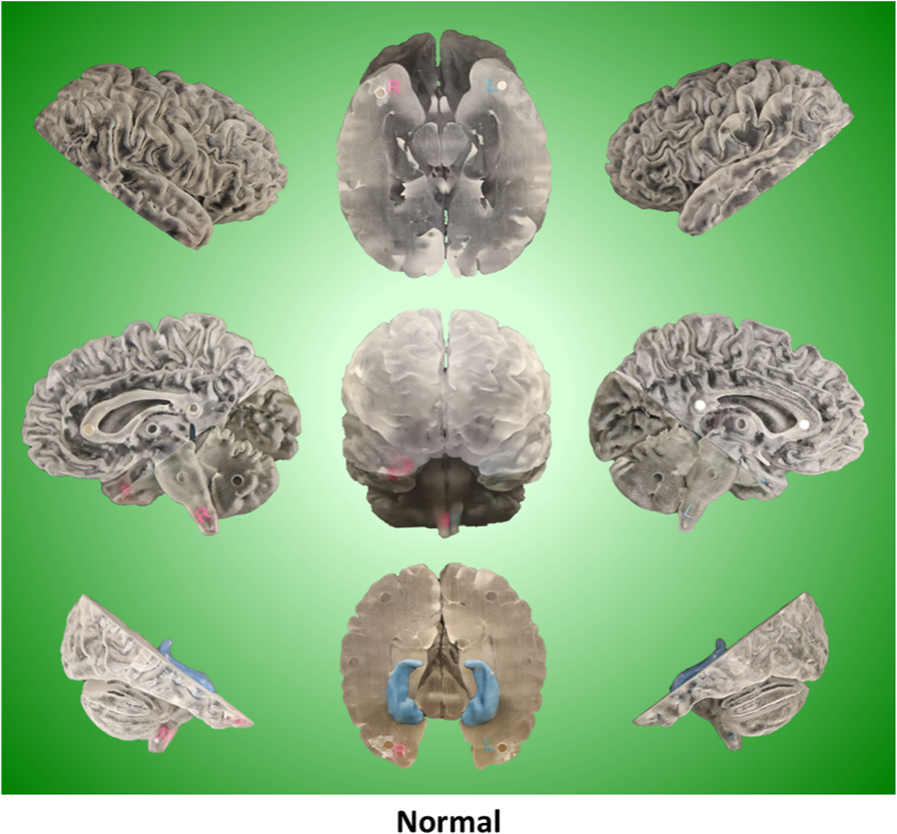
Normal brain 3D–printed model. Photographs are from an early prototype of the normal brain that included labels for the left and right hemispheres, which were omitted in all subsequent models. This particular model was photographed after only several magnets were attached and prior to application of the clear coat spray

**Fig. 11:**
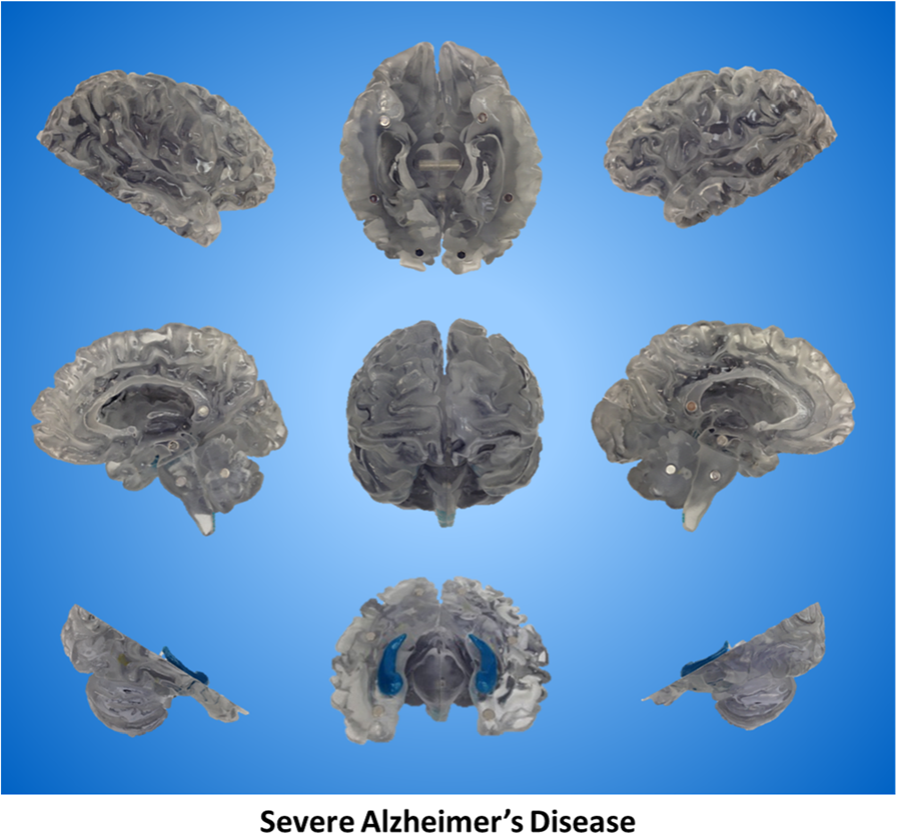
Alzheimer’s brain 3D–printed model. Photographs of the clinically severe (Severe-1) Alzheimer’s brain. Photographs were taken after all magnets were attached and after application of the clear coat spray

The workflow detailed above resulted in the successful production of models for each of the five representative subjects (Figs. 7, 8, 9, 10 and 11) The loss of total brain volume and cortical atrophy with advancing Alzheimer’s disease and age are clearly represented. The anatomic position of the hippocampus and its complex anatomy are easily appreciated by viewing the opaque hippocampus through the semi-transparent media and breaking the model apart to see the hippocampus exposed within the brain. Hippocampal atrophy is evident with advancing disease and age.

**Fig. 12:**
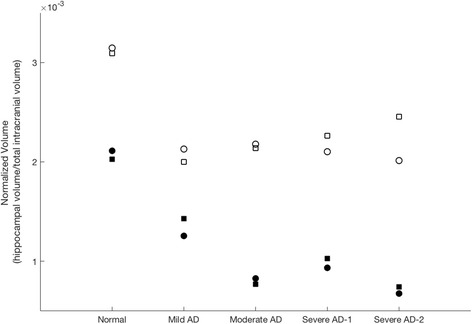
Comparison of manually-smoothed and auto-segmented hippocampal volumes. Hippocampal volumes are normalized to total intracranial volume. Filled circle = manually-smoothed left hippocampal volume, open circle = auto-segmented left hippocampal volume, filled square = manually-smoothed right hippocampal volume, open square = auto-segmented right hippocampal volume. AD = Alzheimer’s disease. Severe AD-1 = clinically severe Alzheimer’s disease. Severe AD-2 = radiologically severe Alzheimer’s disease

The manually segmented models were consistently lower in volume than the auto-segmented models (confidence interval = [−1452–2229], *p* = 2.0 × 10^−6^), reflecting the fact that editing for smoothness removes many more pixels than it adds. The computed values of the two methods were significantly correlated (rho = .85, *p* = .002) (Fig. 12). The manually segmented volumes appeared to decline more with advancing Alzheimer’s disease severity, than did the auto-segmented volumes.

Each model takes approximately 25–35 h for segmentation and CAD work. The cost of a model was approximately $900 USD for printer use, materials and post-processing.

## Discussion

The workflow described in detail here produces life-sized, individualized brain and hippocampal models in subjects with Alzheimer’s disease or other neurologic conditions. 3D–printed models have demonstrated significant educational value in other medical specialties. When 3D–printed models were used during consultation for pediatric patients with congenital heart disease, the patients’ parents and the cardiologists both rated the usefulness of the models very highly and believed they improved communication during the consultation [12].

In a double blind trial of undergraduate medical students, 3D models of cardiac anatomy resulted in higher test scores than cadaveric learning [13]. It is anticipated that these brain models would also serve well as educational aids in a variety of settings including individual patient and family education, medical or neurologic training, patient advocacy or support groups, and public health policy or governmental forums. We believe that holding the models and viewing them will help patients and family members understand Alzheimer’s disease as a tangible physical process and lessen the fear, mystery, and shame often associated with the diagnosis. Construction of these models entails a high investment in editing time, but once created they can be a permanent educational resource.

Limitations of our approach are acknowledged. These models are intended to be useful for demonstration and not diagnosis. Hippocampal volumes resulting from our manual correction protocol are not validated as reliable indicators of Alzheimer’s disease. While it is reassuring that our hippocampal models have computed volumes that clearly decline with increasing Alzheimer’s disease severity, validation would require comparison of our computed volumes with volumes of pathologically dissected specimens and reliability testing would require comparison of volumes determined by multiple operators. Manual hippocampal segmentation has been shown to be as or more reliable than auto-segmentation algorithms; however, our protocol of editing auto-segmented files for smoothness would need validation with a large number of patients and controls, and work-intensive manual segmentation is impractical on a large scale [14]. Also, our present models do not clearly display changes in cortical thickness that may represent an important pathology of Alzheimer’s disease [15]. Finally, our models do not portray other important changes in Alzheimer’s disease such as the distribution of amyloid and tau or altered functional activity of the cerebral cortex.

Future directions in the three-dimensional modeling of Alzheimer’s disease will aim at addressing these limitations. Automated segmentation could be performed with impartial mathematical smoothing of atlas-based algorithms or with algorithms based on shape. Such algorithms have the potential to produce smooth models without time-consuming manual editing. A large number of representative brains could be run through the algorithm to establish age-corrected, disease-specific biomarkers that could be compared to other validated automated procedures. Models with diagnostic importance could then be produced on a patient-by-patient basis. Rigid cortical models could be developed that would allow elimination of white matter with printing, thus allowing better appreciation of cortical thinning and improved visualization of the hippocampus in the brain. We are currently planning the mapping of PiB-PET imaging, Tau-PET-imaging and FDG-PET imaging onto our current brain models to demonstrate additional biomarkers for educational purposes.

## Conclusions

The rising epidemic of Alzheimer’s disease requires educational materials that will increase awareness, facilitate understanding, and stimulate action. We believe that 3D–printed brain models using the workflow explained here are ideal for this purpose.

## Abbreviations

CAD: Computer-aided design
CT: Computerized tomographic
DICOM: Digital imaging and computing in medicine
DRS: Dementia rating scale
EADC-ADNI: European Alzheimer’s Disease Consortium - Alzheimer’s Disease Neuroimaging Initiative
FDG: [(18)F]-fluorodeoxyglucose
GE: General electric
HarP: Harmonized protocol
MP-RAGE: Magnetization-prepared rapid acquisition gradient-recalled echo sequence
MRI: Magnetic resonance imaging
MRML: Medical reality markup language
NIfTI: Neuroimaging informatics technology initiative
NRRD: Nearly raw raster data
PET: Positron emission imaging
PiB: Pittsburgh compound B
ROI: Region of interest
STL: Standard triangle language
